# Germline genetic variants in pheochromocytoma/paraganglioma: single-center experience

**DOI:** 10.1530/EO-22-0091

**Published:** 2023-05-10

**Authors:** José V Lima, Nilza M Scalissi, Kelly C de Oliveira, Susan C Lindsey, Caroline Olivati, Elisa Napolitano Ferreira, Claudio E Kater

**Affiliations:** 1 Department of Medicine, Division of Endocrinology and Metabolism, Universidade Federal de São Paulo (UNIFESP), São Paulo, SP, Brazil; 2 Santa Casa de São Paulo School of Medical Sciences, São Paulo, SP, Brazil; 3 Fleury Group, São Paulo, SP, Brazil

**Keywords:** SDHB, VHL, RET, NF1, TMEM127, MAX, SDHD

## Abstract

Pheochromocytoma and paragangliomas (PPGLs) are rare neuroendocrine tumors carrying 25–40% pathogenic germline gene variants (PGVs). We evaluated clinical, laboratory, and germline molecular profile of 115 patients with pathologic (14 patients were relatives from 8 different families recruited for genetic survey) confirmed PPGL followed in our institution. Patients with classic MEN2A/MEN2B phenotypes and at-risk relatives underwent direct analysis of *RET* proto-oncogene, and the remaining had samples submitted to complete next-generation sequencing aiming 23 PPGL-related genes: *ATM, ATR, CDKN2A, EGLN1, FH, HRAS, KIF1B, KMT2D, MAX, MDH2, MERTK, MET, NF1, PIK3CA, RET, SDHA, SDHAF2, SDHB, SDHC, SDHD, TMEM127, TP53,* and* VHL*. We also developed a clinical judgment score (CJS) to determine the probability of patients having a potentially hereditary disease. The resulting genetic landscape showed that 67 patients (58.3%) had variants in at least one gene: 34 (50.7%) had exclusively pathogenic or likely pathogenic variants, 13 (19.4%) had pathogenic or likely pathogenic variants and variant of undetermined significance (VUS), and 20 (29.8%) carried only VUS. PGVs were found in *RET* (*n* = 18; 38.3%), *VHL* (*n* = 10; 21.3%), *SDHB* and *NF1* (*n* = 8; 17% each), and *MAX*, *SDHD*, *TMEM127,* and *TP53* (*n* = 1; 2.1% each). Direct genetic testing disclosed 91.3% sensitivity, 81.2% specificity, and 76.4% and 93.3% positive predictive value (PPV) and negative predictive values (NPV), respectively. The CJS to identify patients who would not benefit from genetic testing had 75% sensitivity, 96.4% specificity, and 60% and 98.2% PPV and NPV, respectively. In summary, the landscape of PPGL germline gene variants from 115 Brazilian patients resulted in slightly higher prevalent pathogenic and likely pathogenic variants, especially in the *RET* gene. We suggest a CJS to identify PPGL patients who would not require initial genetic evaluation, improving test specificity and reducing costs.

## Introduction

Pheochromocytomas and paragangliomas (PPGLs) are rare neuroendocrine tumors that produce, store, and secrete catecholamines (Sutton *et al.* 1981, Karagiannis *et al.* 2007, Neumann *et al.* 2019, Al Subhi *et al.* 2022). The outdated general ‘rule of 10%’, which was formerly applied for genetic behavior for PPGL (Sutton *et al.* 1981, Bravo & Tagle 2003) also used to include inheritance. However, this rule no longer stands. Regarding genetic behavior, 25–40% of PPGL carriers have germline variants (Lenders *et al.* 2014, Garcia-Carbonero *et al.* 2021); for paragangliomas (PGLs) alone, there is a 50% chance of finding a pathogenic variant (PV) (Antonio *et al.* 2020), a rate greater than that for any other human neoplasm (Toledo *et al.* 2017).

Currently, nearly 70% of PPGLs result from germline or somatic PVs in a single driver gene. To date, there are more than 20 genes related to PPGLs, and more new genes are being discovered (7–10).

The genomic characteristics of PPGLs allow us to allocate them into three clusters according to their transcriptional signature (Buffet *et al.* 2017, Toledo *et al.* 2017, Jiang *et al.* 2020, Garcia-Carbonero *et al.* 2021, Cascon *et al.* 2023, Giminez-Roqueplo *et al.* 2023).

Cluster 1 includes genes related to the Krebs cycle. Characteristic genes are *SDHA, SDHAF2, SDHB, SDHC, SDHD, FH,MDH2, GOT2, IDH1, SCLC25A11, EPAS1,* and *VHL*. The presence of germline or somatic PVs in these genes leads directly or indirectly to deregulaton in *HIF1a* and *HIF2a*, generating pseudohypoxia and increasing angiogenesis and cell proliferation (Buffet *et al.* 2017, Toledo *et al.* 2017, Jiang *et al.* 2020, Garcia-Carbonero *et al.* 2021).

Cluster 2 has signature activation of MAP kinase signaling pathways. The following genes stand out: *NF1, RET, HRAS,* and *TMEM127* (9–11).

Cluster 3 (identified by The Cancer Genome Atlas) has WnT pathways based on a transcriptional signature, which also increases the risk of metastatic PPGLs.

The aim of this study was to evaluate the germline molecular profile of a cohort of patients with PPGL from a single center in Sao Paulo, SP, Brazil.

## Material and methods

### Study design and participants

This prospective and retrospective study included 115 PPGL patients followed at the Endocrine Division, Universidade Federal de Sao Paulo (Unifesp), Brazil, who were evaluated due to the presence of (i) adrenal/extra-adrenal tumors and/or (ii) secondary arterial hypertension and/or (iii) because they had first-degree relatives with PPGLs. Fourteen patients were relatives from eight different families recruited for genetic survey.

Clinical, laboratory, and genetic data were collected between February 2000 and December 2019. The inclusion criterion was pathologic confirmation of PPGL.

The study was approved by the Ethics and Research Committee of Unifesp. All patients or their legal responsible signed an informed written consent form. Patients were distributed into two groups, named as ‘potentially hereditary’ (group 1) and as ‘apparently sporadic’ (group 2). The characteristics that defined the potentially hereditary cases are shown in Table 1. Patients presenting at least one of the characteristics suggestive of hereditary behavior entered group 1 (*n* = 91 patients) and those who did not present any of those characteristics comprised group 2 (*n* = 24 patients).

**Table 1:** Clinical, laboratory, and imaging features suggestive of germline pathogenic variants.

	Potentially hereditary
Associated diseases	Medullary thyroid carcinoma, primary hyperparathyroidism, neurofibromatosis, middle ear tumors, central nervous system hemangioblastoma, pancreatic neuroendocrine tumor, gastrointestinal stromal tumor (GIST), clear cell renal carcinoma, pituitary adenoma, Hirschsprung’s disease
Clinical, laboratory, and imaging features	Skin pigmentation (*cafe-au-lait* spots), precocious puberty, marfanoid habitus, mucosal neuromas, retinal angiomas, renal and/or pancreatic cysts, polycythemia, intestinal ganglioneuromatosis, megaesophagus, megacolon, and amyloidotic cutaneous lichen

Additionally, we developed a clinical judgment score (CJS) (Table 2) to determine the probability of a patient having a potentially hereditary disease.

**Table 2:** Clinical judgment score.

Clinical score	Points
<20 years old	Mandatory genetic test
Any paraganglioma (PGL), regardless of age Classic clinical picture of NF1, VHL, MEN2A, MEN2B, or familial PGL syndrome	
20–45 years of age	3
Family history of PPGL	3
Presence of bilateral masses	2
Presence of metastasis	1
Total	9

We accessed the Fleury laboratory database of uncharacterized patients who underwent a genetic panel by next-generation sequencing (NGS) for PPGL from January 2018 through July 2022 and collected data from 105 unrelated patients. The Fleury Group operates in the four main regions of Brazil – Northeast, Midwest, Southeast, and South. Patients underwent the exam because they had a PPGL.

### Data collection

The following data were collected from each patient: age; sex; family history of PPGL; presence of syndromic presentation; presence of comorbidities; use of antihypertensive medications; measurements of urinary and plasma metanephrines and catecholamines, vanillylmandelic acid (VMA), and chromogranin A; imaging findings of the abdomen, chest, and neck; pathologic results; and genetic testing (germline genetic panel by NGS for PPGL or Sanger genetic screening of the *RET* proto-oncogene, if the case had a typical MEN2A or 2B presentation). Somatic assessment was not performed at initial assessment and will not be discussed in this paper. The results of the genetic tests were classified as pathogenic, likely pathogenic, or variant of undetermined significance (VUS) according to the American College of Medical Genetics (ACMG) (Richards *et al.* 2015). Benign findings were not reported.

### Genetic testing

Patients with the classic MEN2A or MEN2B phenotypes and their at-risk relatives underwent direct analysis of the *RET* proto-oncogene at the Laboratory of Molecular and Translational Endocrinology at Unifesp. DNA extraction and PCR amplification of genomic DNA were prepared from blood leukocytes according to standard protocols. Oligonucleotide primers for the amplification of different *RET* exons were designed at intronic sequences flanking exons 8, 10, 11, 13, 14, 15, and 16. PCRs were performed in a final volume of 25 µL containing 20 mM Tris–HCl (pH 8.4), 50 mM KCl, 1.5 mM MgCl_2_, 0.2 mM deoxynucleotide triphosphate, 1 U Taq polymerase, and 1 mM specific primers and using 100 or 200 ng of genomic DNA as input. Genomic DNA was denatured for 3 min at 94°C before 35 cycles of 1 min each at 94°C, at 65°C, and at 72°C, followed by 5 min at 72°C in a thermocycler (Corbett Research, Qiagen Ltd, Sydney, Australia). Following PCR, the amplicon sizes were analyzed on a 1.8% agarose gel, and the products were visualized by staining with GelREd® (Uniscience Ltd, Osasco, SP, Brazil). PCR products were purified with a commercial kit (Life Technologies, Inc.) and sequenced using an automated system employing fluorescent dye terminators (ABI Prism 3100, Applied Biosystems) (Lindsey *et al.* 2012).

Variant analyses were performed based on the medullary thyroid carcinoma guidelines by the American Thyroid Association (Wells *et al.* 2015, Thomas *et al.* 2019) and scored according to the evidence evaluated (prediction, literature, and information deposited in Arup, ClinVar, PubMed, and Varsome).

Patients who did not have the classic MEN2A or MEN2B phenotypes had their samples submitted to a genetic panel by NGS at Fleury Group laboratory with the complete sequencing of all coding region and adjacent flanking regions of 23 genes related to PPGL. The analysis included identification of pathogenic genetic variants and copy number variations by NGS.

DNA was extracted from peripheral blood leukocytes using the QIAsymphony DNA Mini Kit according to the manufacturer's recommendations: 200 ng of DNA were used for library preparation following the protocol for Sophia Genetics' Custom Bundle Solution. Capture-based target enrichment was performed on pools of up to eight samples using a custom panel for analysis of full-coding sequencing and flanking splice sites of 23 PPGL-related genes.

Sequencing was performed using a NextSeq® 500/550 Mid Output Kit v2 (300 cycles) on a NextSeq® platform (Illumina). The mapping of reads to the human reference genome GRCh37/hg19 and the calling of variants were performed by a custom bioinformatics pipeline, and the annotation and interpretation of variants were performed on the Sophia DDM® platform (Sophia Genetics, Boston, USA; https://www.sophiagenetics.com/).

Average coverage for targeted region was 722.5× of depth (minimum of 357× and maximum of 1788×). For the majority of the samples, we had ≥99.98% of the target region with minimum of 50× of depth of coverage (minimum 99.3% and maximum of 100%).

We used the scores from the association of the ACMG (American College of Medical Genetics and Genomics) and the ACGS (American Clinical Genomic Science) Best Practice Guidelines for Variant Classification 2019 for the analyses.

We used Emedgene® software with all information relevant to the criteria and scored the reads according to the evidence evaluated (prediction, literature, and information deposited in PubMed, Arup, ClinVar, and Varsome).

The 23 genes analyzed were *ATM, ATR, CDKN2A, EGLN1, FH, HRAS, KIF1B, KMT2D, MAX, MDH2, MERTK, MET, NF1, PIK3CA, RET, SDHA, SDHAF2, SDHB, SDHC, SDHD, TMEM127, TP53,* and* VHL.*

The genetic analysis of 47 of the 105 controls (44.7%) was performed by the same platform Sophia DDM®, whereas the remaining 58 (55.3%) were investigated using the platform Twist Bioscience®; the average coverage for targeted region was 752.5× of depth (minimum of 411.75× and maximum of 1065×). For the latter 58 samples, we had 100% of the target region with minimum of 50× of depth of coverage (minimum 99.6% and maximum of 100%).

### Hormonal testing

Hormonal evaluation for the biochemical diagnosis of PPGL was performed with 24-h urinary metanephrines, 24-h urinary catecholamines, 24-h urinary VMA, plasma metanephrines, and plasma catecholamines. As of 2014, and in accordance with the Endocrine Society's PPGL Guideline (Lenders *et al.* 2014), we only performed tests for plasma and/or 24-h urinary fractionated metanephrines and VMA, whereas urinary and plasma catecholamines were discontinued.

### Statistical testing

All statistical analyses were performed using IBM SPSS Statistics for Windows, Version 25.0 (Released 2019; IBM Corp, Armonk, NY, USA). Descriptive analysis included absolute (*n*) and relative (%) frequencies and bar graphs of qualitative variables and summary measures (mean, standard deviation, median, minimum, and maximum). Inferential analysis included the association test using the chi-square or Fisher's exact test and the logistic regression-stepwise forward method and receiver operating characteristic (ROC) curve analysis to evaluate possible cutoff points for quantitative variables, complemented by the calculation of sensitivity and specificity. A significance level of 5% was adopted for all tests.

## Results

### Patients, age, clinical presentation, time of presentation

The median age of the patients was 44.5 years (range 14–79); 74 patients were women and 41 were men.

Ninety-one patients (79.1%) satisfied the criteria that suggested PPGL was potentially hereditary (group 1) and 24 (20.9%) did not (group 2). In groups 1 and 2, 63.4% and 66.6% of the patients were women.

The association of systemic arterial hypertension (SAH) + paroxysms and orthostatic hypotension was present in 16 (34%) of genetically confirmed cases, followed by SAH + paroxysms in 3 (6.4%), orthostatic hypotension in 4 (8.5%), isolated SAH in 6 (12.8%), SAH + orthostatic hypotension in 8 (17%), isolated paroxysms in 3 (6.4%), and orthostatic hypotension + paroxysms in 1 (2.1%). Six (12.8%) of the patients were normotensive.

### Distributions of germline variants

The genetic landscape of the 115 PPGL patients submitted to the genomic study showed that 67 (58.3%) had a germline variant in at least 1 of the 23 PPGL-related genes studied: 34/67 (50.7%) had exclusively pathogenic or likely PVs, 13 (19.4%) pathogenic or likely PVs and VUS, and 20 (29.8%) carried only VUS. This group included germline variants in the following genes: *RET* (18; 38.3%), *VHL* (10; 21.3%), *SDHB,* and *NF1* (8; 17% each*)* and *MAX*, *SDHD*, *TMEM127,* and *TP53* (1; 2.1% each). The VUS group (*n* = 42 germline variants (GVs)) included GVs in the following genes: *ATM* (9; 21.4%), *KMT2D* (6; 14.2%), *MERTK* (5; 11.9%), *SDHC*,* MET*,* RET*, and *FH* (3; 7.1% each)*, SDHA* and* NF1* (2; 4.7% each), and *ATR,CDKN2A*, *SDHAF2, KIF1B,MDH2,* and *MAX* (1; 2.4% each).

**Figure 1:**
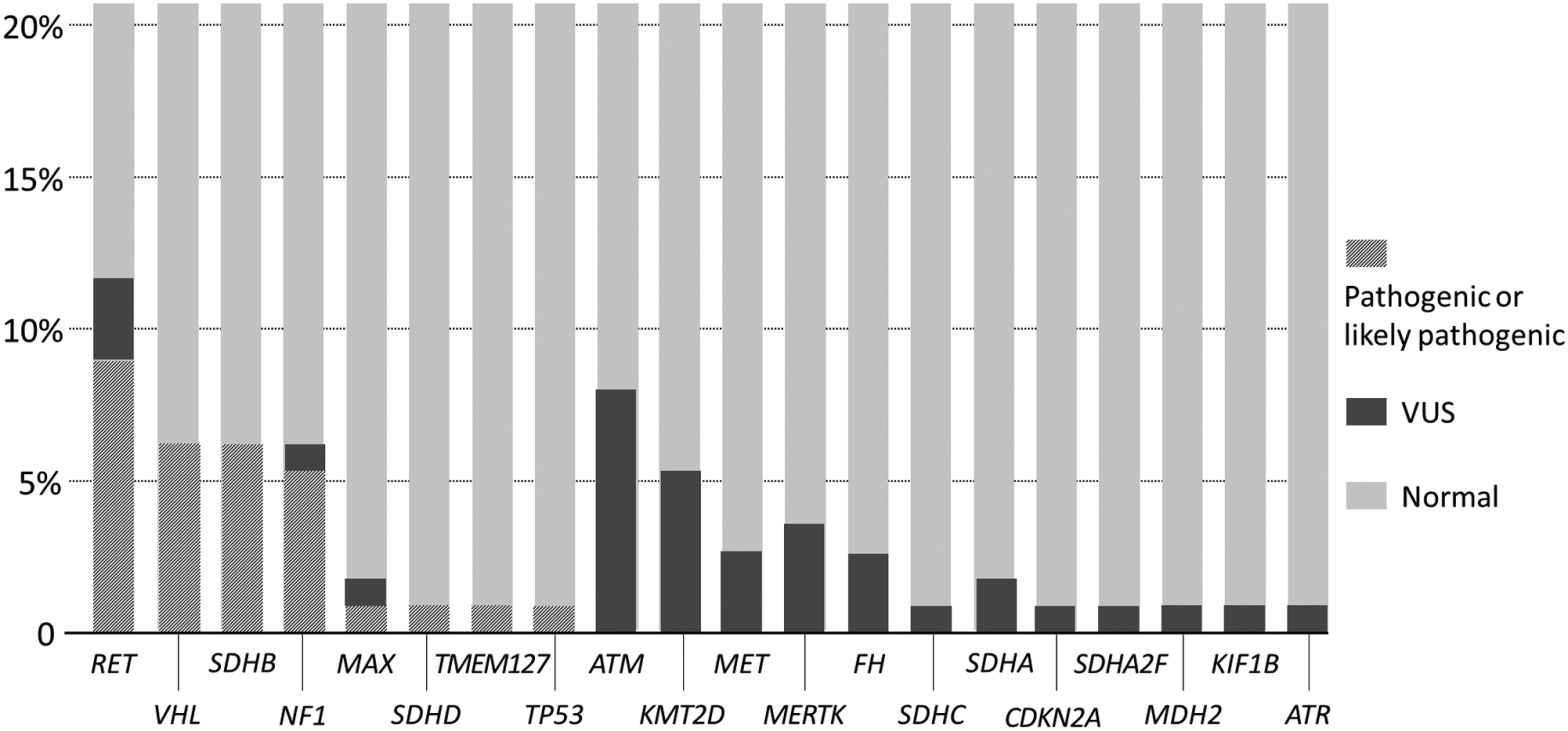
Genetic distribution of 101 PPGL patients (excluding 14 relatives) and 105 PPGL controls according to ACMG criteria.

**Figure 2:**
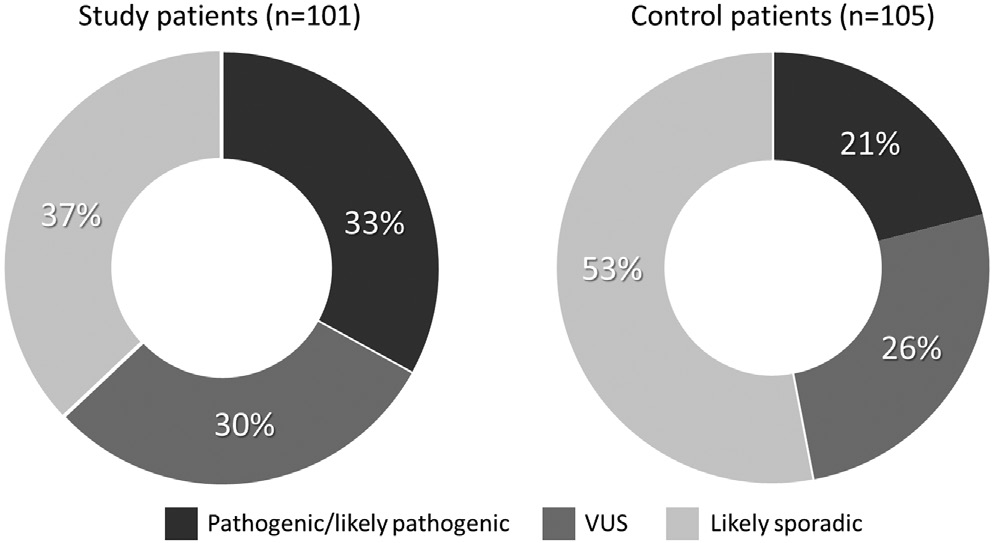
Distribution of 72 germline variants in 101 PPGL patients (excluding 14 relatives).

Since 14 subjects from 115 PPGL patients were relatives from eight different families, we recalculated germline variants distribution exclusively from the 101 PPGL index cases. This information is presented in Figs. 1 and 2.

A table with VUS cases is provided as a supplementary material (see section on supplementary materials given at the end of this article). We did not perform segregation or functional studies of VUS cases, but all patients who were followed up in the Adrenal Unit at Unifesp were reassessed regularly.

In the sample of 105 PPGL individuals from the Fleury Group lab, PVs were present in 22 (21%) individuals and VUS in 35 (33.3%), whereas 56 (53.3%) subjects did not have any variants (Fig. 1). The PV distribution was as follows: 5 (22.7%) in each *SDHB*, *VHL*, and *RET*, followed by 2 (9%) in each *SDHD* and* MERTK*, and 1 (5.7%) in each *ATM*, *NF1*, *SDHA*, and *TMEM127*. The VUS distribution was as follows: 8 (22.8%) *ATM*, 5 (14.3%) *KMT2D*, 4 (11.4%) in each *MERTK* and *SDHA*, 2 (5.7%) in each *NF1, FH*, *RET*, and *CDKN2A*, and 1 (2.8%) in each *ATR, KIF1B, SDHC, TMEM127, SDHB,* and* MET.*

Regarding age distribution, none of the patients were in the first decade of life. For patients in the second decade of life, 50% had PV in the *RET* gene, 25% in *VHL,* and 25% in *SDHB* genes. For patients in the third decade of life, the incidence of PV was highest for the *VHL* and *SDHB* genes (28.6% each), followed by *RET*, *MAX*, and *NF1* genes (14.2% each). For patients in the fourth decade of life, the incidence of PV was highest for the *RET* gene (36.3%), followed by *VHL* and *SDHB* (18.2% each) and the *SDHD*, *TMEM127*, and *NF1* genes (9.1% each). For patients in the fifth decade of life, PV in the *RET* gene predominated (61.5%), followed by *VHL* and *NF1* (15.4%, each) and the *SDHB* gene (7.7%). For patients in the sixth decade of life, PV in the *NF1* gene predominated (66.6%), followed by the *RET* and *VHL* genes (16.7% each). For patients in the seventh decade of life, 50% had PV in *SDHB* and 50% in *VHL* genes.

Table 3 shows the general characteristics of the PPGL cohort and Table 4 shows the genetic (B) characteristics of the PPGL cohort.

**Table 3:** General characteristics of the genetic PPGL cohort.

Family	Positive cases		Age at diagnosis (years)	PPGL^a^ (with metastasis^g^)	Hormonal profile^b^	Imaging^c^	Years of follow-up
	With PPGL	Without PPGL					
01	1	–	22	Unilateral Pheo	NMN + MN	CT	14
	3		45	Bilateral Pheo		MRI + MIBG	
			19				
			14				
02	3	–	44	Unilateral Pheo	NMN + MN	MRI + MIBG	12
			40				
			32				
	2		44	Bilateral Pheo			
			52				
03	2	–	49	Unilateral Pheo	NMN + MN	CT + MIBG	14
			18				
04	1	–	50	Unilateral Pheo	NMN + MN	MRI + MIBG	10
05	1	–	35	Unilateral Pheo	NMN + MN	MRI + MIBG	10
06	1	–	17	Unilateral Pheo	MN	MRI + MIBG	10
07	1	1	42	Bilateral Pheo	NMN + MN	MRI + MIBG	11
08	1	–	45	Unilateral Pheo	MN	MRI + MIBG	12
09	1	–	34	Bilateral Pheo	MN	MRI + MIBG	3^f^
10	1	1	37	Bilateral Pheo	NMN + MN	MRI + MIBG	5
11	3	–	45	Bilateral Pheo	NMN	MRI + MIBG	15
			40				
			18				
12	1	5	38	Unilateral Pheo	Nonfunct.^f^	MRI + MIBG	4
13	1	–	21	Abdominal PGL ^#^	NMN	MRI + MIBG	17
	1		17				13
14	1	–	48	Unilateral Pheo	NMN	MRI + MIBG	14
15	1	–	23	Unilateral Pheo	NMN	MRI + MIBG	15
16	1	1	33	Bilateral Pheo	NMN	MRI + MIBG	13
17	1	1	38	Bilateral Pheo	NMN	MRI + MIBG	7
18	1	2	62	Neck PGL^#^	Nonfunct.	MRI + MIBG	15
19	1	–	50	Unilateral Pheo	NMN	MRI + MIBG	14^f^
20	1	8	17	Abdominal PGL^#^	NMN	MRI + MIBG	5
	1		39	Neck PGL	Nonfunct.		7
21	1	–	23	Abdominal PGL ^#^	NMN	MRI + MIBG	12
22	1	–	28	Abdominal PGL	NMN	MRI + MIBG	12
23	1	–	32	Abdominal PGL	NMN	MRI + MIBG	13
24	1	–	17	Neck PGL	Nonfunct.	MRI + MIBG	4
25	1	–	59	Unilateral Pheo	NMN + MN	MRI + MIBG	14
26	1	–	33	Unilateral Pheo ^#^	NMN + MN	MRI + MIBG	24^f^
27	1	–	52	Unilateral Pheo	Nonfunct.	MRI + MIBG	13
28	1	–	31	Unilateral Pheo	NMN + MN	MRI + MIBG	13
	1		53	Bilateral Pheo	Nonfunct.		
29	2	–	49	Unilateral Pheo	NMN + MN	MRI + MIBG	11^f^/15
			29				
30	1	–	60	Unilateral Pheo	NMN + MN	MRI + MIBG	15
31	1	1	39	Neck PGL	Nonfunct.	MRI + MIBG	8
32	1	–	22	Abdominal PGL	NMN + MN	MRI	12
33^d^	1	1	32	Unilateral Pheo	NMN + MN	CT + MIBG	21
		1					
Total	47	22	Median= 35 years (14–62)	Unilateral = 21 Bilateral = 15 Neck PGL = 4 Abdominal PGL = 7 ^#^with metastasis= 6	NMN+MN= 23 NMN= 14 MN= 3 Nonfunct. = 7	MRI + MIBG= 41 CT + MIBG=4 CT=1 MRI= 1	Median = 13 years (3–24)

^a^PPGL, pheochromocytoma (= Pheo; adrenal)/paraganglioma (= PGL; extra-adrenal).

^b^Hormonal profile: MN, metanephrines; NMN, normetanephrines.

^c^Imaging: CT, computerized tomography, MIBG, metaIodo-benzyl-guanidine; MRI, magnetic resonance imaging.

^d^Index case and one relative (without PPGL) with PV in the *TMEM127* and *TP53* genes and one relative with PV in the TMEM127 gene only.

^e^*MAX, SDHD,* and *TMEM127/TP53.*

^f^Death.

^g^Metastasis

Nonfunct., nonfunctioning.

**Table 4:** Genetic characteristics of the PPGL cohort.

Family	Patients with PPGL	Gene	Familial syndromes	Other tumors	Nucleotide (NT)	Protein
01	4	*RET*	MEN2A	MTC / PHP	c.1900T>G	p. Cys634Gly
02	5	*RET*	MEN2A	MTC / PHP	c.1900T>G	p. Cys634Gly
03	2	*RET*	MEN2A	MTC	c.1901G>A	p. Cys634Tyr
04	1	*RET*	MEN2A	MTC	c.1901G>A	p. Cys634Gly
05	1	*RET*	MEN2A	MTC	c.1900T>C	p. Cys634Arg
06	1	*RET*	MEN2B	MTC	c.2753T>C	p. Met918Thr
07	1	*RET*	MEN2A	MTC	c.1859G>C	p. Cys620Ser
08	1	*RET*	MEN2B	MTC	c.2753T>C	p. Met918Thr
09	1	*RET*	MEN2B	MTC	c.2753T>C	p. Met918Thr
10	1	*RET*	MEN2A	MTC/PHP	c.1900T>C	p. Cys634Arg
11	3	*VHL*	VHL2A	Pancreatic NET (1)/retinal angioma (2)/cerebellum HB (2)	c.496G>T	p. Val166Phe
12	1	*VHL*	VHL2C		c.256C>T	p. Pro86Ser
13	2	*VHL*	VHL2C		c.467A>G	p. Tyr156Cys

14	1	*VHL*	VHL2B	Pancreatic NET/CCRC	c.233A>G	p. Asn78Ser
15	1	*VHL*	VHL2C		c.239G>T	p. Ser80Ile
16	1	*VHL*	VHL2B	Pancreatic NET+retinal angioma+cerebellum HB+CCRC	c.499C>T	p. Arg167Trp
17	1	*VHL*	VHL2A	Cervical HB	c.293A>G	p. Tyr98Cys
18	1	*SDHB*	PGL4	PHP	c.293G>A	p. Cys98Tyr
19	1	*SDHB*	PGL4		c.591del	p. Ser198Alafs*22
20	2	*SDHB*	PGL4	GIST (1)	c.137G>T	p. Arg46Leu
21	1	*SDHB*	PGL4		Del involving exon 1, identified by CNV	
22	1	*SDHB*	PGL4		c.3G>A	p. Met1?
23	1	*SDHB*	PGL4		Del involving exon 1, identified by CNV	
24	1	*SDHB*	PGL4		c.724C>A	p. Arg242Ser
25	1	*NF1*	NF1		c.1260+1G>A	p. (?)
26	1	*NF1*	NF1		c.5487_5490 dup	p. Gly1831Profs*11
27	1	*NF1*	NF1		c.4537C>T	p. Arg1513*
28	2	*NF1*	NF1		c.1527+4_1527+7 del (Del of 4 NT at position +4 (intron 13)	Affects a NT from the splicing donor site that possibly alters the processing of messenger RNA by skipping exon 13
29	2	*NF1*	NF1		c.6999 +2 T>G	p. (?)
30	1	*NF1*	NF1		c.7248_7256del	p. Tyr2417_Ala2419del
31	1	*SDHD*	PGL1		c.196A>G	p. Met66Val
32	1	*MAX*			Del involving exons 1 through 4*, identified by CNV	
33**	1	*TMEM127 / TP53*	-/Li-Fraumeni	Breast Ca+pancreatic Ca	c.117_120 delc.1010 G>A	p. Ile41Argfs*39 p. Arg337His
Total	47	*RET= 18* *VHL= 10* *SDHB= 8* *NF1= 8* *Other^$^= 3*	MEN2A=15 MEN2B= 3 VHL2A=4 VHL2B=2 VHL2C=4 PGL4=8 PGL1=1			

(1), one affected patient; (2), two affected patients.

CCRC, clear cell renal carcinoma; HB, hemangioblastoma; MTC, medullary thyroid carcinoma; PHP, primary hyperparathyroidism.

### Topographic distribution and metastases

In the confirmed hereditary patients, 34% had bilateral PPGL, 34% had right adrenal PPGL, 15% had retroperitoneal PGL, 8.5% had left adrenal PPGL and 8.5% had neck PGL. Among the bilateral PPGL cases, we identified germline PVs in *RET* (56.2%), *VHL* (37.5%), and *NF1* (6.3%) genes (Table 3).

Six patients (12.7%) with germline PV had metastatic PPGL: four had PV in *SDHB*, one in *VHL*, and one in *NF1* genes. The most frequent sites of metastasis were lymph nodes (*n* = 5), bone (*n* = 5), liver (*n* = 4), blood vessels (*n* = 4), and lungs (*n* = 3). Three patients with metastatic PPGL (two with PV in *SDHB* and one with PV in *NF1*) died because of tumor progression. Table 3 also shows individual imaging procedures for PPGL diagnosis and time of follow-up for each patient.

### Clinical judgment score

Using the criteria for direct genetic testing, we found a sensitivity of 91.3%, specificity of 81.2%, positive predictive value (PPV) of 76.4%, negative predictive value (NPV) of 93.3%, and accuracy of 85.2%.

When the CJS was used to identify patients who did not benefit from the genetic test using a cutoff of ≥4 points, we found a sensitivity of 75%, specificity of 96.4%, PPV of 60%, NPV of 98.2%, and accuracy of 95%.

This clinical score developed from our cohort of patients was useful to exclude those who would not benefit from genetic testing.

The following characteristics were statistically significant: age, bilateral masses, classic PPGL-related disease phenotypes, and family history of PPGL. Metastasis and topography (adrenal or extra-adrenal) were not statistically significant. On logistic regression, the association with the greatest statistical power (to determine whether to perform the genetic test) was age ≤45 (*P* = 0.016 with odds ratio (OR) of 5.9 (1.4–25)), classic PPGL-related disease phenotypes (*P* < 0.001 with OR of 189 (34.4–1,038.3)), and paraganglioma (*P* < 0.001 with OR of 8.3 (1.0–39.3)). Family history, bilateral masses, and metastasis were not statistically significant in the logistic regression model.

The age cutoff of 45.5 years disclosed a sensitivity of 70.2% and a specificity of 54.4% (area under the curve of 0.694; *P* < 0.001) for the diagnosis of potentially genetic PPGL, as per ROC curve analysis.

### Correlation of germline variants and metanephrines

Hormone production (urine and/or plasma metanephrines and normetanephrines) correlated with germline PV, as shown in Table 3. Note that cases with PVs in the *RET, NF1, MAX,* and* TMEM127* genes had a predominant adrenergic profile, whereas cases with PVs in *VHL* and *SDHB* had an exclusive noradrenergic profile.

## Discussion

The incidence of certain affected genes in PPGL is higher according to the population studied. The Chinese population, for instance, has fewer germline variants than Europeans. *SDHx* genes predominate among the European population (Bausch *et al.* 2017, Jiang *et al.* 2020).

Our study evaluated the profile of germline PPGL gene variants from 115 patients from a single reference center in São Paulo, Brazil. This population sample originated in part from an outpatient clinic that is specialized in MEN; this may have produced some reference bias, resulting in a higher PV frequency of the *RET* gene; on the other hand, it is also possible that the prevalence of *RET* gene PV in the Brazilian population is actually higher, as was also observed with the control sample from the Fleury Group databases, which encompasses patients from the four major regions of Brazil. Some reports have demonstrated that the *RET* PV profile may vary according to geographical area (Pena *et al.* 2009, Pena *et al.* 2011, Maia *et al.* 2014, Bausch *et al.* 2017, Cunha *et al.* 2017, Maciel *et al.* 2019, 2021). Further studies should be carried out to clarify this issue. Neumann *et al.* highlighted a greater incidence of PV in the *VHL* gene in the first decade of life, which decreases in the third decade of life but remains one of the most important genes found. From the second decade of life onward, the incidence of PV in the genes of the *SDHx* complex began to increase, assuming a greater proportion in cases of PGL and metastasis. The incidence of *RET* gene variants is important around the third decade of life but is less than those of the *VHL* and *SDHx* genes, highlighting the characteristic of bilateral disease and virtual absence of metastases. *NF1* gene variants stand out in the fourth and fifth decades of life. (Neumann *et al.* 2019).

The distribution in our population sample was different: *RET* was the most prevalent gene with PV in the second, fourth, and fifth decades of life*,* whereas* NF1* was the most prevalent gene with PV in the sixth decade of life. The *SDHB* and *VHL* genes were present in all decades of life.

The pathogenic or likely PVs found in this study were comparable to those reported in the medical literature. Rare variants, however, were found in the *SDHB*, *VHL*, and *NF1* genes, such as *SDHB*: c.137G>T in exon 2, c.293G>A in exon 4, and c.724C>A in exon 7; *VHL*: c.467A>G and c.496G>T, both in exon 3; and *NF1*: c.5487_5490dup resulting in frameshift in exon 37, c.1527+4_1527+7del in intron 13, and c.6999+2T>G in intron 46. We also found a unique association of PV in the *TMEM127* and *TP53* genes (R337H) in a 47-year-old patient who developed bilateral metastatic breast cancer, meningioma of the CNS, and pancreatic cancer; her 20-year-old daughter is a carrier of the same variants who did not develop PPGL thus far but faced bilateral breast cancer and meningioma of the CNS and her 5-year-old granddaughter carries only the *TMEM127* gene variant and currently has no clinical or laboratory evidence of PPGL.

The patient with PV c.5487_5490dup in the *NF1* gene developed bone, liver, and lung metastases at 22 years after PPGL resection and died after developing severe febrile neutropenia following therapy with ^131^I-mIBG; to our knowledge, this is the first NF1-associated PPGL case reported in which metastasis developed only 20 years later.

There is a strong genetic determinism in PPGL. Clinical features at the initial diagnosis of PPGL may suggest germline variants, allowing for grouping of such cases as potentially hereditary, especially in the presence of typical phenotypic conditions, as mentioned before. It is generally agreed that all PPGLs must be submitted to genetic evaluation (Jiang *et al.* 2020, Garcia-Carbonero *et al.* 2021, Cascon *et al.* 2023, Gimenez-Roqueplo *et al.* 2023); however, when the availability of genetic NGS panels is greatly limited, as in Brazil, we suggest the use of a CJS to define which individuals should be initially exempt from genetic testing while maintaining a permanent follow-up, with the need for genetic testing being reassessed periodically. Although our CJS had excellent specificity, NPV, and accuracy, it must be validated prospectively. On the other hand, the association of the presence of genetic variants with age <45 years, the presence of PPGL-related genetic syndromes, and topography (adrenal/extra-adrenal) strongly indicates that patients with such characteristics should undergo genetic testing.

The clinical characteristics employed to direct NGS research in our patients were sufficient to guarantee helpful coverage for the main related genes, achieving good sensitivity with acceptable specificity and useful NPV.

As per the ACMG, 33% of our cohort of 101 index cases was classified as pathogenic/likely pathogenic, 30% as VUS, and 37% as probably sporadic. Therefore, 63% carry some germline variant, which is slightly higher than other published series. All VUS cases are periodically reassessed to determine whether they will change category; thus far, however, all such cases in this study remain VUS, a percentage that agrees with the literature.

Although *ATM* gene variants were significantly in our cohort, there is no current evidence between *ATM* variants and the presence of PPGL, making this finding of unknown clinical significance.

When compared to the control group (patients from the Fleury Lab database), we noticed a higher rate of PV. We did not have access to clinical or imaging characteristics of the Fleury Group of patients, once they were uncharacterized, but it is possible that they were not properly selected for genetic testing; conceivably, the use of the CJS would have reduced the number of sporadic cases. We believe that use of the CJS would make genetic testing more judicious and cost-effective for public and private health systems.

In summary, we describe the landscape of PPGL germline gene variants from a large sample of Brazilian patients in which pathogenic and likely PVs were highly prevalent, especially in the *RET* gene. We also suggest a CJS to identify PPGL patients who would not require initial genetic evaluation, improving test specificity and reducing costs.

## Supplementary Materials

Supplementary Table 1

## Declaration of interest

There is no conflict of interest that could be perceived as prejudicing the impartiality of this research.

## Funding

This project has been partially supported by the Fleury Lab Group (grant numbers NP-335 and NP- 334). This work did not receive any specific grant from any funding agency in the public.
