# Supplementary material for: Germline genetic variants in pheochromocytoma/paraganglioma: single-center experience
Source: Endocr Oncol. 2023 May 10;3(1):e220091. doi: 10.1530/EO-22-0091 (PMC10388674; doi:10.1530/EO-22-0091)
Supplement: Supplementary Table 1 [file supplementary_table_1.pdf]

Case	Gene	Transcript effect	Protein	Classification	Allele frequency	
					gnomAD (%)	abraOm (%)
1	*RET*	NM_020630.4: c.1900T>C	p.(Cys634Arg)	Pathogenic	0.0012	0
	SDHA	NM_001294332.1: c.1306A>G	p.(Ile436Val)	VUS	0.001	0
2	*VHL*	NM_000551.3: c.496G>T	p.(Val166Phe)	Pathogenic	0	0
	NF1	GRCh37/hg19:Chr17(29.559.075_29.559.223)*dup Duplication involving exon 25	-	VUS	?	?
	SDHC	GRCh37/hg19:Chr1(161.284.086_161.332.440)*dup Duplication involving exons 1 to 6	-	VUS	?	?
3	*VHL*	NM_000551.3: c.496G>T	p.(Val166Phe)	Pathogenic	0	0
	SDHC	GRCh37/hg19:Chr 1(161.284.086_161.332.440)*dup Duplication involving exons 1 to 6	-	VUS	?	?
4	*NF1*	NM_000267.3: c.6999+2T>G	p.(?)	Likely pathogenic	0	0
	MERTK	NM_006343.2: c.475T>A	p.(Ser159Thr)	VUS	0.0035	0
5	*NF1*	NM_000267.3: c.6999+2T>G	p.(?)	Likely pathogenic	0	0
	MERTK	NM_006343.2: c.475T>A	p.(Ser159Thr)	VUS	0.0035	0
6	*NF1*	NM_000267.3: c.1527+4_1527+7del		Likely pathogenic	0	0
	ATM	NM_000051.3: c.2428A>G	p.(Lys810Glu)	VUS	0.0014	0
7	*MAX*	GRCh37/hg19:Chr14(65.543.084_65.569.172)*del Deletion involving the exons 1 to 4	-	Likely pathogenic	?	?
	KMT2D	NM_003482.3: c.3508C>G	p.(Pro1170Ala)	VUS	0	0
8	*VHL*	NM_000551: c.233A>G	p.(Asn78Ser)	pathogenic	0	0

	ATR	NM_001184: c.7518A>G	p.(Glu2506=)	VUS	0	0
	KMT2D	NM_003482: c.2214C>G	p.(Ser738=)	VUS	0	0
9	*MERTK*	NM_006343.2: c.2720C>T	p.(Thr907Ile)	VUS	0.001	0
10	*ATM*	NM_000051: c.5185G>C	p.(Val1729Leu)	VUS	0.01	0.0821
11	*ATM*	NM_000051.3: c.749G>A	p.(Arg250Gln)	VUS	0.0049	0.0821
12	*RET*	NM_020630 : c.1759C>T	p.(Arg587Trp)	VUS	0.0008	0
13	*SDHAF2*	GRCh37/hg19:Chr11(61.213.303_61.213.654)* dup Duplication involving exon 4	-	VUS	?	?
14	*KMT2D*	NM_003482.3: c.8046+4del	(p.(?))	VUS	0	0
15	*KMT2D*	NM_003482.3: c.1369C>T	p.(Pro457Ser)	VUS	0.00044	0.0854
16	*RET*	NM_020630: c.1651A>T	p.(Ile551Phe)	VUS	0	0
17	*FH*	NM_000143: c.151C>T	p.(Arg51Trp)	VUS	0.001	0
	ATM	NM_000051: c.946T>C	p.(Tyr316His)	VUS	0.002	0
	MDH2	NM_001282403: c.502T>G	p.(Ser168Ala)	VUS	0.007	0
18	*ATM*	NM_000051.3: c.4148C>T	p.(Ser1383Leu)	VUS	0.004	0
	MAX	NM_001320415.1: c.164C>T	p.(Ser55Leu)	VUS	0	0
	NF1	NM_000267.3: c.5843A>T	p.(Gln1948Leu)	VUS	0.008	0
19	*KMT2D*	NM_003482: c.3134C>T	p.(Pro1045Leu)	VUS	0.001	0
	MET	NM_000245: c.2342A>C	p.(Glu781Ala)	VUS	0.001	0
20	*MET*	NM_000245.3: c.40C>T	p.(Leu14Phe)	VUS	0.008	0.1642
21	*ATM*	NM_000051.3: c.1516G>T	p.(Gly506Cys)	VUS	0.003	0
	MET	NM_000245.3: c.508A>C	p.(Ser170Arg)	VUS	0.0007	0
22	*CDKN2A*	NM_058195.3: c.160C>A	p.(Arg54Ser)	VUS	0.006	0

23	*MERTK*	NM_006343.2: c.232G>A	p.(Val78Ile)	VUS	0.0039	0.0821
24	*ATM*	NM_000051.3: c.7187C>G	p.(Thr2396Ser)	VUS	0.018	0
25	*ATM*	NM_000051.3: c.6537T>G	p.(Ile2179Met)	VUS	0.005	0.2463
26	*ATM*	NM_000051.3: c.6825A>G	p.(Ile2275Met)	VUS	0	0
27	*KIF1B*	NM_015074.3: c.430-4A>G	(p.(?))	VUS	0.0003	0
28	*FH*	NM_000143.3: c.50C>T	p.(Ala17Val)	VUS	0.0084	0
	SDHA	NM_001294332.1: c.278T>C	p.(Leu93Pro)	VUS	0	0
29	*VHL*	NM_000551.3: c.499C>T	p.(Arg167Trp)	Pathogenic	0.001	0
	FH	NM_000143.3: c.4T>C	p.(Tyr2His)	VUS	0.006	0
30	*TMEM 127*	NM_001193304.2: c.117_120del	p.(Ile41Argfs*39)	Pathogenic	0.001	0
	TP53	NM_000546.5: c.1010G>A	p.(Arg337His)	Pathogenic	0	0
	RET	NM_020630.4: c.1234G>A	p.(Val412Met)	VUS	0.0007	0
31	*RET*	NM_020630.4: c.2753T>C	p.(Met918Thr)	Pathogenic	0.0004	0
	MERTK	NM_006343.2: c.1840C>G	p.(Leu614Val)	VUS	0	0
32	*SDHB*	GRCh37/hg19: Chr1(17.380.333_17.380.624)* del Deletion involving exon 1	-	Pathogenic	?	?
	KMT2D	NM_003482.3: c.9662C>A	p.(Thr3221Asn)	VUS	0.02	0.0821
33	*VHL*	NM_000551.3: c.496G>T	p.(Val166Phe)	Pathogenic	0	0
	SDHC	GRCh37/hg19: Chr1(161.284.086_161.332.440)*dup Duplication involving exons 1 to 6	-	VUS	?	?

Title: VUS allele frequencies in gnomAD and abraOm from 33 patients with PPGL

* We evaluated copy number variations (CNVs) that comprise 1 or more exons, but genomic coordinates of breakpoints for CNVlike variants are not described.

? Not described in gnomAD and abraOm
